# Spit-Tacular Science: Collaborating With Undergraduates on Publishable Research With Salivary Biomarkers

**DOI:** 10.3389/fpsyg.2019.00562

**Published:** 2019-03-21

**Authors:** Katherine L. Goldey, Erin E. Crockett, Jessica Boyette-Davis

**Affiliations:** ^1^Department of Psychology and Behavioral Neuroscience, St. Edward's University, Austin, TX, United States; ^2^Department of Psychology, Southwestern University, Georgetown, TX, United States

**Keywords:** undergraduate research, saliva, biomarkers, hormones, collaboration

Training in physiological methods substantially increases students' competitiveness for graduate school, medical school, and multiple career paths. For example, when asked to rank the value of various types of research skills among applicants to PhD programs, neuroscience graduate program directors ranked background knowledge in the student's application area first, closely followed by bench skills (Boyette-Davis, 2018). Physiological measures are also increasingly incorporated within personality and social psychology, especially when studying relationship processes or social stress and marginalization (e.g., Smyth et al., 2013). This suggests that faculty can best serve students who want to study biology-behavior interactions in graduate school or in their careers by providing them with research experience in physiological methods. However, collaborating with undergraduates on physiological research is often challenging given extensive training and equipment costs associated with many biopsychological methods (e.g., fMRI, PET), especially when the goal is to publish with undergraduate students as authors.

Here, we present a model for collaborative faculty-undergraduate research involving human salivary biomarkers. We outline opportunities presented by faculty-student research with salivary biomarkers, strategies for addressing challenges of this approach, and concrete recommendations for success. Although our recommendations are based on our experiences as faculty at small liberal arts universities (1,400 to 4,000 undergraduates), many of our suggestions could apply to other types of institutions, especially regional comprehensives to larger institutions. We focus on the research design, data collection, and data analysis stages, given that best practices for writing and publishing with undergraduates are similar to those in other domains of psychology and will be addressed elsewhere in this issue (see also Jones et al., 2006; Burks and Chumchal, 2009).

## Opportunities

A wide variety of hormones (e.g., testosterone, cortisol, CCK), cytokines, opioids, and immunoglobulins can be measured via saliva using commercially-available enzyme immunoassay (or ELISA) kits. Salivary biomarkers can thus address questions of intrinsic interest to students and are relevant to multiple subdisciplines of psychology. Examples of topics students have investigated under our supervision include how testosterone predicts pain responding in women (Archey et al., 2018), how thinking about competition affects testosterone, how non-suicidal self-injury is related to opioid levels, associations between sexual compliance and cortisol levels (Hartmann and Crockett, 2016), and the effects of support processes on cortisol reactivity (Crockett et al., 2017). These topics often link biomarkers to physical and mental health, which is becoming increasingly relevant as researchers think more critically about health and wellness. Moreover, biomarker research is very popular with students: in our labs, we each run one to three studies a year in collaboration with 5–10 students total; we each turn away 10–20 additional students due to limited resources.

Working with salivary biomarkers teaches students important theoretical skills (e.g., principles of behavioral endocrinology, how ELISAs work), research skills (e.g., data collection with human participants), and practical laboratory skills (e.g., pipetting, creating serial dilutions). This approach encourages students to make connections between social and natural sciences and attracts an interdisciplinary group of students, including Behavioral Neuroscience, Psychology, Biology, and Kinesiology majors with future plans ranging from PhD programs to health professions. For these reasons, research with salivary biomarkers is ideal for educating students while also providing practical preparation for careers. Compared to blood samples, saliva samples are far more feasible for working with undergraduates, given that they pose low (to no) biohazard risk and do not require invasive techniques.

## Challenges and Solutions

Although less expensive than some physiological methods, research with salivary biomarkers requires equipment (plate reader, centrifuge, plate mixer, pipettes, and optionally a plate washer) and disposable materials (ELISA kits, pipette tips, etc.). Startup costs can be as low as $6k with used equipment or up to $10–15k with new equipment, and yearly costs range from $3-5k. Compared with neuroimaging, which requires significant grant contributions and thousands of dollars per study, salivary biomarker research is feasible (albeit challenging) on a tight budget. We have funded our research programs by supplementing departmental funding with small grants from Psi Chi or professional societies and by purchasing secondhand equipment. These strategies carry the added bonus of developing undergraduates' grant-writing and budgeting skills.

A second challenge, particularly when producing publishable salivary biomarker research at smaller universities, is obtaining a sufficient sample size. For example, when measuring testosterone in females, salivary measures underestimate hormone-behavior associations compared to blood measures, necessitating large sample sizes (e.g., 30–50 participants per experimental condition or group; Granger et al., 2004). Additionally, some health conditions and the use of hormonal contraceptives may confound results, requiring sample sizes robust enough to allow for the exclusion of some participants or the addition of multiple control variables to statistical models. We have addressed these issues by planning for data collection to span two to three semesters, which presents its own challenges when students need to complete a project before graduation. We encourage students to include at least one relevant non-hormonal outcome, such as survey responses or behavioral data; this allows for students to present preliminary results at on-campus or regional conferences and to have the potential for publication in the event of null biomarker results. Larger teams of students can also facilitate recruitment as each student can recruit from different student organizations or courses. Even with modest incentives (extra credit offered at professors' discretion and raffles for gift cards), our highly motivated students have succeeded in recruiting 100 participants in two semesters.

## Recommendations

So you've decided you want to collaborate with undergraduates on salivary biomarker research with the goal of publication – how do you start? The first step toward publication is for undergraduates to produce high-quality research. Here, we discuss best practices with a focus on processes unique to salivary biomarkers.

1. Undergraduate students must be trained in sampling issues and ethical considerations associated with salivary biomarker collection before drafting Institutional Review Board (IRB) proposals. For example, most biomarkers require querying participants' medication use, nicotine use, sleep/wake habits, relevant health conditions, and relevant social behaviors (e.g., relationship status as a covariate for testosterone) via questionnaires (Kirschbaum and Hellhammer, 1994; Smyth et al., 2013; van Anders et al., 2014). Additionally, sample collection is typically limited to specific times of day (e.g., 2 h after eating for CCK; Ekström et al., 2019). All analytes we have tested show a lag time to respond to social stimuli, such that the timing of samples in experimental studies must be carefully planned (Dickerson and Kemeny, 2004; van Anders et al., 2014; Archey et al., 2018). Reading and discussing a paper that reviews methodology for the biomarkers of interest is a useful way to introduce students to these issues, and students are often intrinsically interested to learn how everyday behaviors such as waking time or social variables such as relationship status affect hormones. We require students to draft the IRB protocol and to prepare a research proposal (Introduction and Method sections), which teaches scientific writing skills and helps prepare for the goal of publication.
2. Second, students must be trained on the collection and storage of saliva samples. Collection is relatively easy, as biomarkers can be collected via passive drool into tubes or via salivette (a sterile piece of dental cotton about 2 in long), depending on the analyte. Undergraduates can practice providing instructions for saliva collection to one another, and students can gain leadership experience by training new lab members.
3. The assay process is the most involved in terms of training. In this phase, we each employ different strategies to balance students' need to practice bench skills with the need to obtain reliable results suitable for publication with student co-authors. If mentors want students to complete all steps of the assay, including pipetting the plate, students should ideally be given the opportunity to run a test assay if funding for one extra kit is available. Otherwise, students can practice pipetting with water and a non-antibody coated plate and watch an experienced student pipet an assay before completing an assay themselves. While students complete an assay, the mentor should oversee each step; this supervision means that the process takes longer but helps maintain consistency. Alternatively, the mentor can pipette the plate while students assist in other ways – by centrifuging and organizing samples, operating the plate mixer and reader, etc. Regardless of the strategies used, the assay process is an excellent opportunity for students to gain hands-on research skills, practice troubleshooting when the process does not work as expected, and learn about how ELISAs work via the principle of competitive binding. This firsthand knowledge of the assay process is useful to students when constructing the Materials subsection of an eventual publication.
4. When analyzing data with biomarkers, two important considerations are necessary to achieve publishable results. Biomarker variables should be screened for outliers prior to analysis, and a selection of the most important biomarker confounds/covariates should be included in statistical models. Learning to account for these variables is an invaluable opportunity for students.

## The Value of Cross-Campus Collaboration: A Case Study

The above best practices can be challenging to balance against teaching and service responsibilities at smaller institutions, and equipment costs might be prohibitive for some institutions. Cross-campus collaborations provide an ideal way to share resources and time commitments as well as knowledge and expertise. This expertise is especially useful for new faculty in the process of establishing their labs, or for faculty who are new to salivary biomarker research and were not trained on these methods in graduate school. Collaborative approaches also allow faculty to apply their various expertise in combination with specific student interests. For example, in our most recent publication on associations between testosterone and pain responses in women, JBD contributed her expertise on sex differences in the neurobiology of pain, KLG shared guidelines for salivary testosterone data collection, and EEC led the assay process. Ultimately, our collaboration resulted in a publication (Archey et al., 2018), a national conference presentation, and a travel award for the undergraduate first author, now a neuroscience PhD student.

There were several advantages to this collaborative approach. It allowed for resources to be pooled, students to access different faculty mentors, and faculty to share the responsibility and time commitment associated with training students. Combining multiple faculty members' expertise also meant more varied and interdisciplinary perspectives to provide feedback on the manuscript. There are some important logistical considerations with cross-campus collaborations, some relevant to any human subjects research (e.g., where does IRB oversight rest?), others specific to biomarker research (e.g., transporting samples on ice in a cooler between campuses), and some basic issues such as travel time. Finding collaborators or transporting samples may be more difficult in locations where other universities are not in close proximity.

Even when physical proximity makes close collaborations difficult, it is still possible to form cross-campus collaborations. Recent developments of sites like Study Swap (https://christopherchartier.com/) and Psi Chi's Network for International Collaborative Exchange (NICE) program (https://www.psichi.org/page/Res_Opps#.XEnzSKlME8Y) connect universities across the globe, allowing researchers to post resources they can offer as well as research needs they have. These provide interesting opportunities specific to biomarkers. Often when running experiments with salivary biomarkers, researchers have more time from participants than they need because of the lag time for many biomarkers to respond to social stimuli. Timing samples necessitates having surveys completed, even when the self-reported information is not essential to the research question. As a result, collected self-report data from our participants is often something we can offer in exchange for access to potentially eligible participants at universities where human participant pools are larger than university demands.

## Further Reading and Resources

For overviews of salivary cortisol and testosterone methodology (saliva collection and storage, sample timing, confounds/covariates, etc.), see Kirschbaum and Hellhammer (1994), Smyth et al. (2013), and van Anders et al. (2014).

Instructions we use for saliva collection for cortisol (Salivette) and testosterone (passive drool), as well as the slides we use to teach students about ELISAs for cortisol and testosterone are in the Supplementary Material files.

## Author Contributions

KG, EC, and JB-D conceived and outlined the article. KG wrote the first draft of the article. EC and JB-D wrote sections of the article and revised the article. All authors read and approved the submitted version.

### Conflict of Interest Statement

The authors declare that the research was conducted in the absence of any commercial or financial relationships that could be construed as a potential conflict of interest.
