# Supplementary material for: Spit-Tacular Science: Collaborating With Undergraduates on Publishable Research With Salivary Biomarkers
Source: Front Psychol. 2019 Mar 21;10:562. doi: 10.3389/fpsyg.2019.00562 (PMC6437038; doi:10.3389/fpsyg.2019.00562)
Supplement: Supplementary file 1 [file Presentation_1.pptx]

## Slide 1
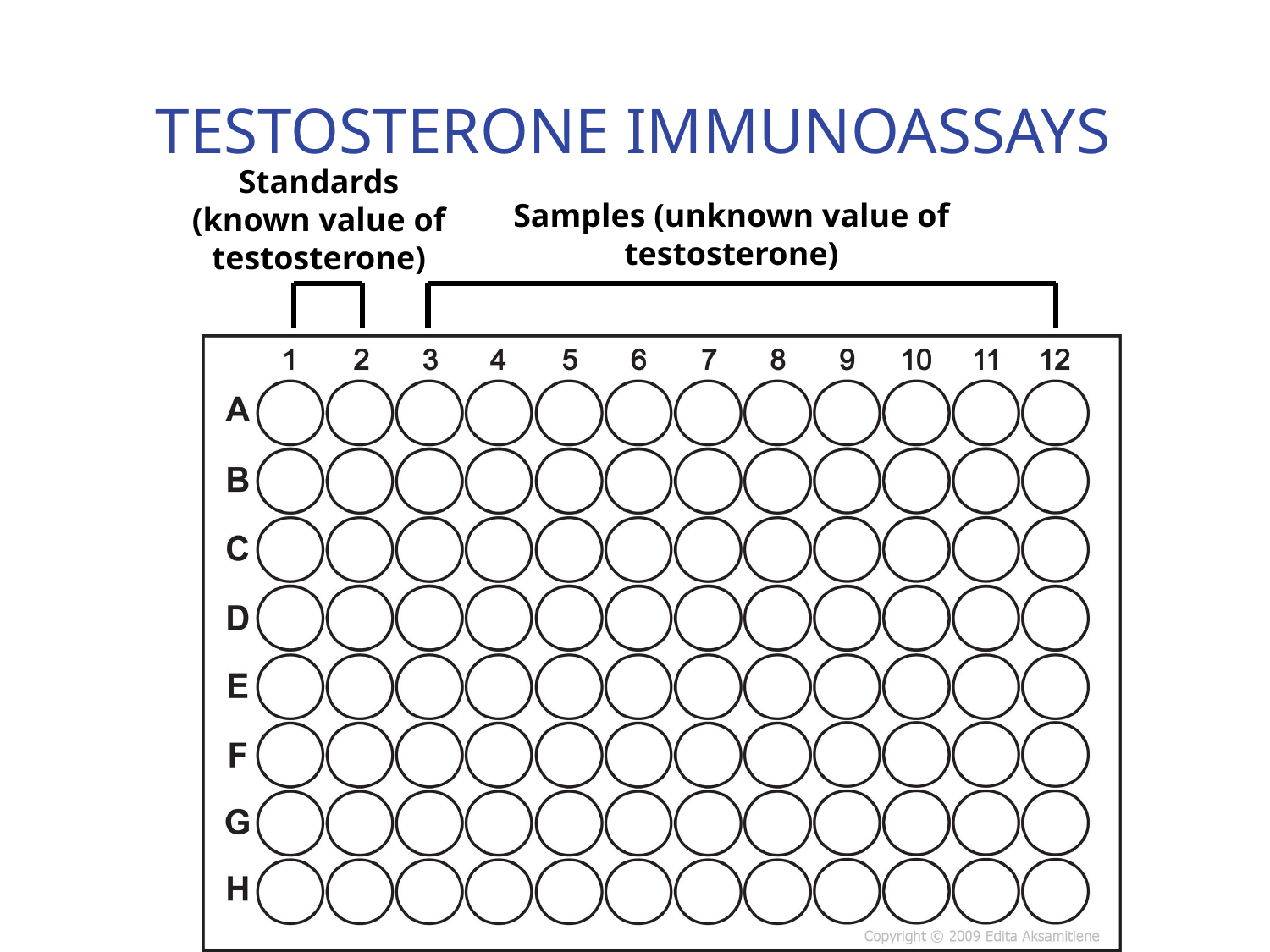

# TESTOSTERONE IMMUNOASSAYS
Standards (known value of testosterone)
Samples (unknown value of testosterone)

## Slide 2
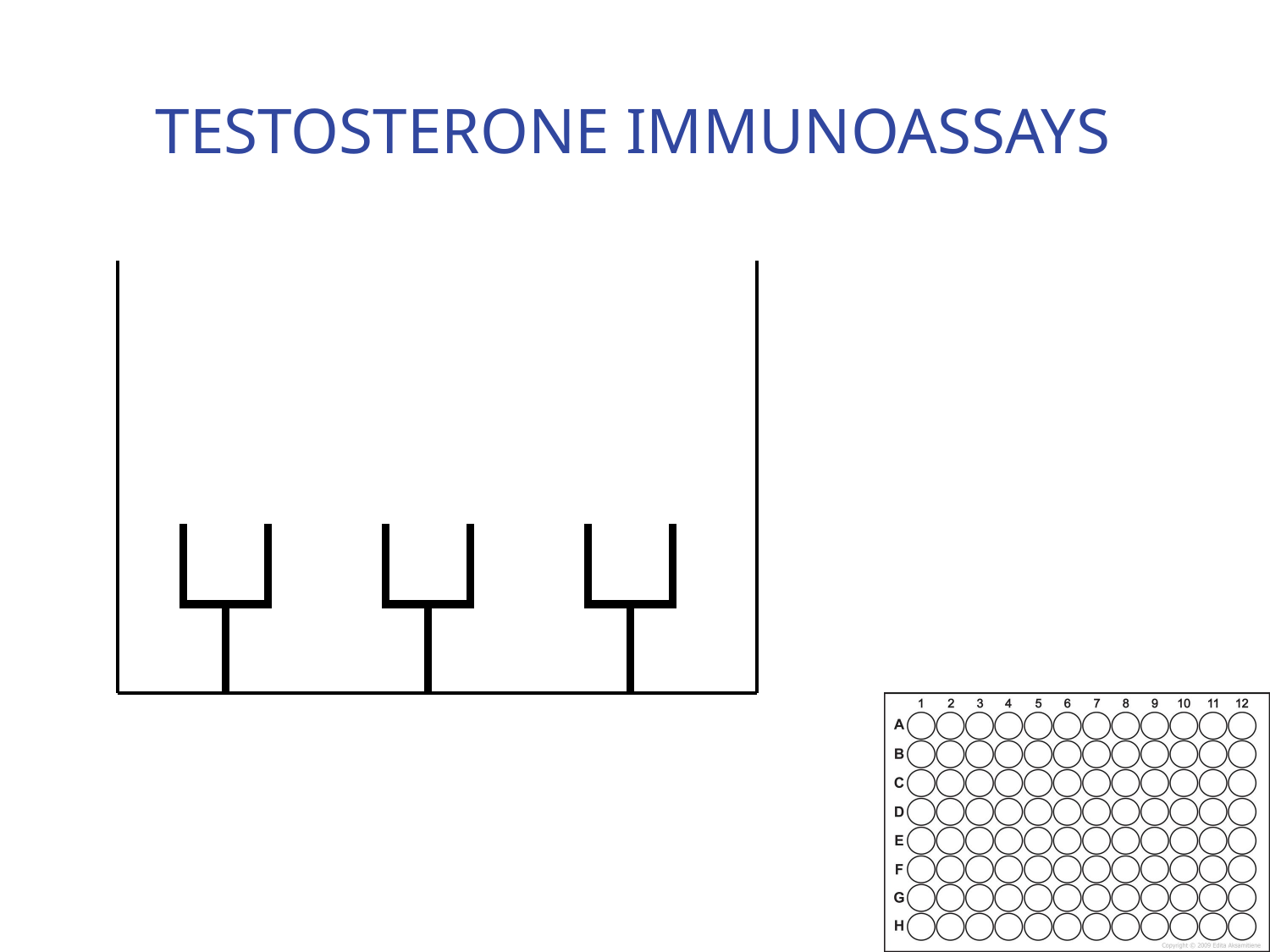

# TESTOSTERONE IMMUNOASSAYS

## Slide 3
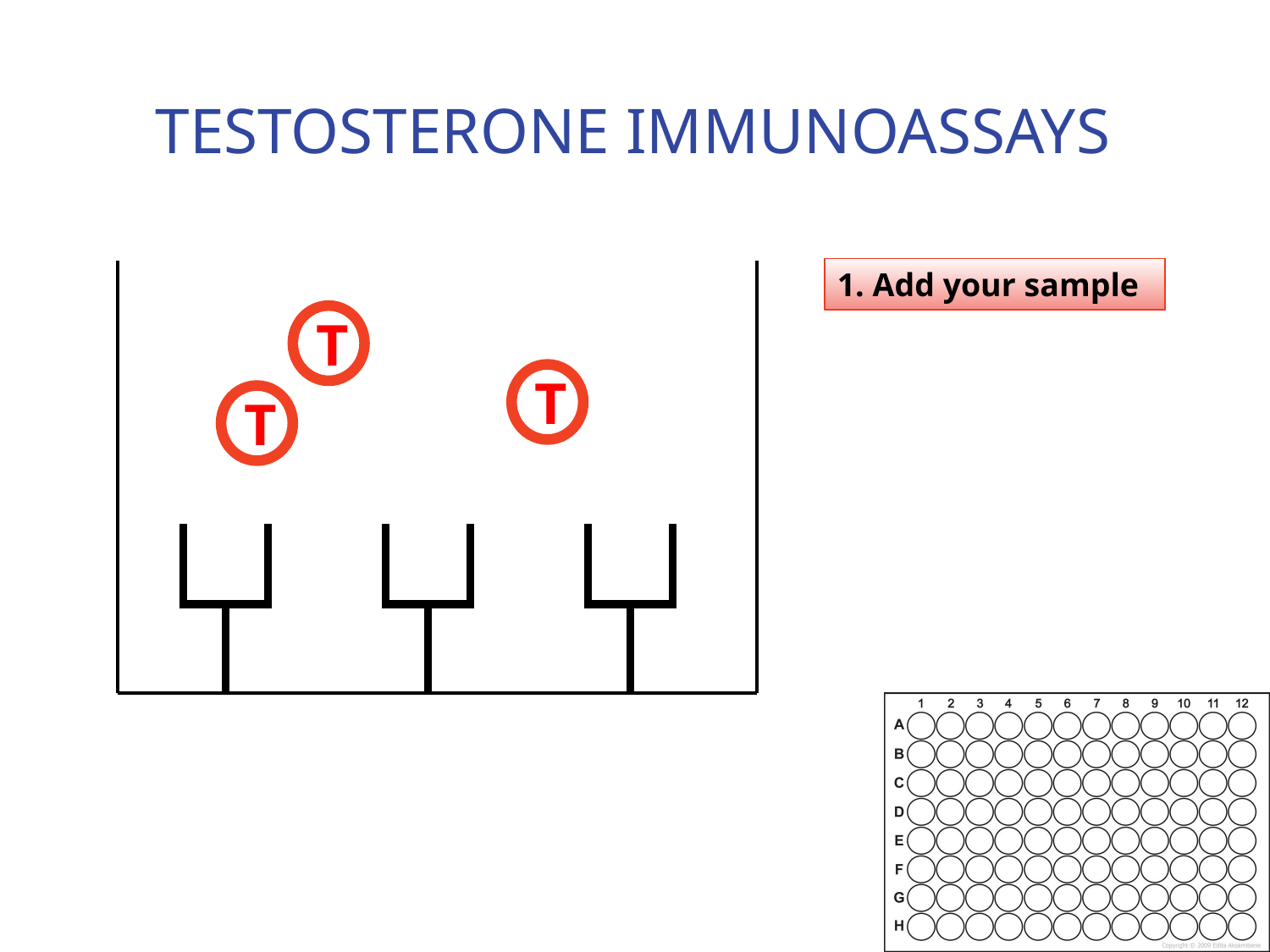

# TESTOSTERONE IMMUNOASSAYS
1. Add your sample
T
T
T

## Slide 4
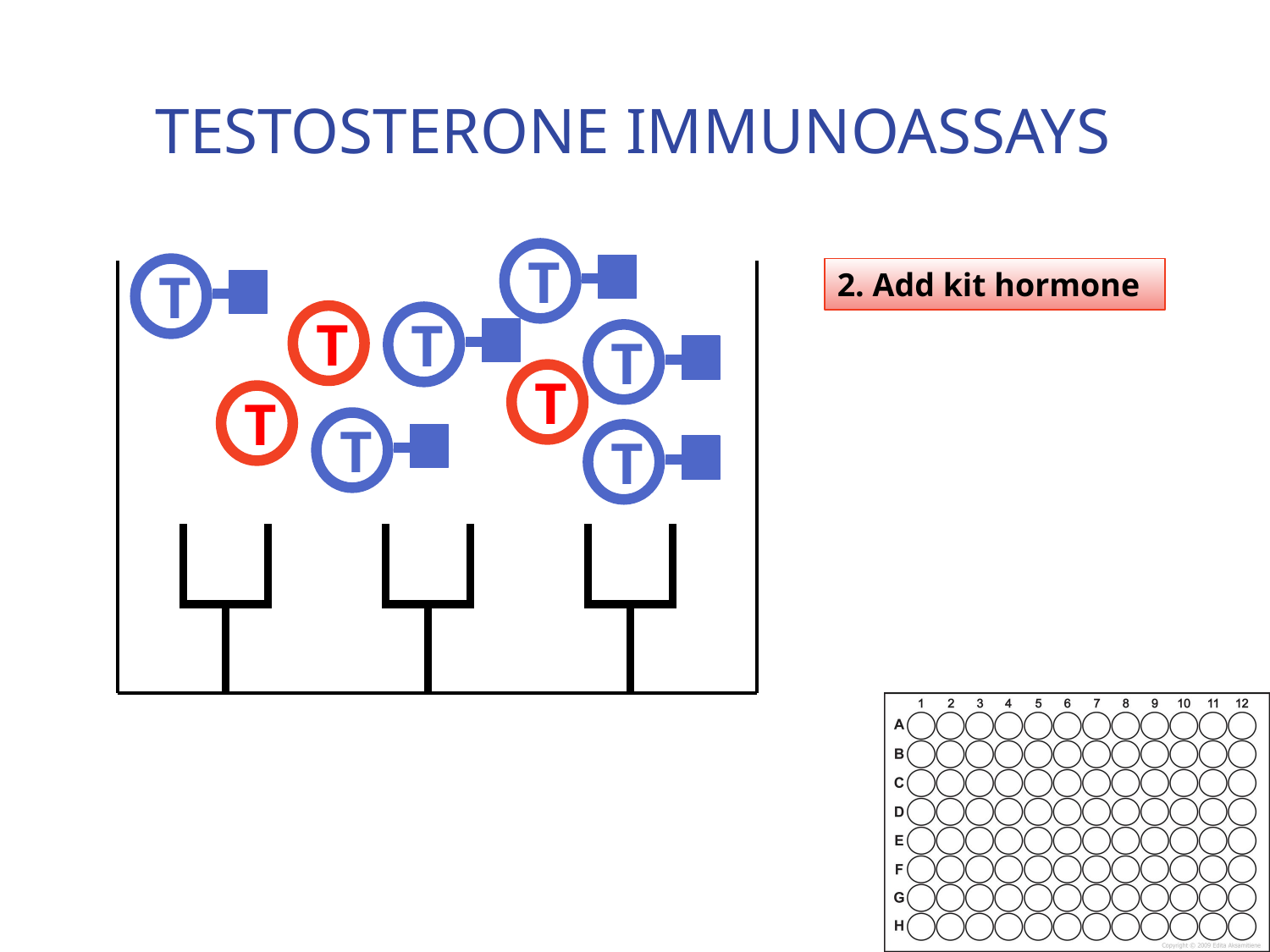

# TESTOSTERONE IMMUNOASSAYS
T
2. Add kit hormone
T
T
T
T
T
T
T
T

## Slide 5
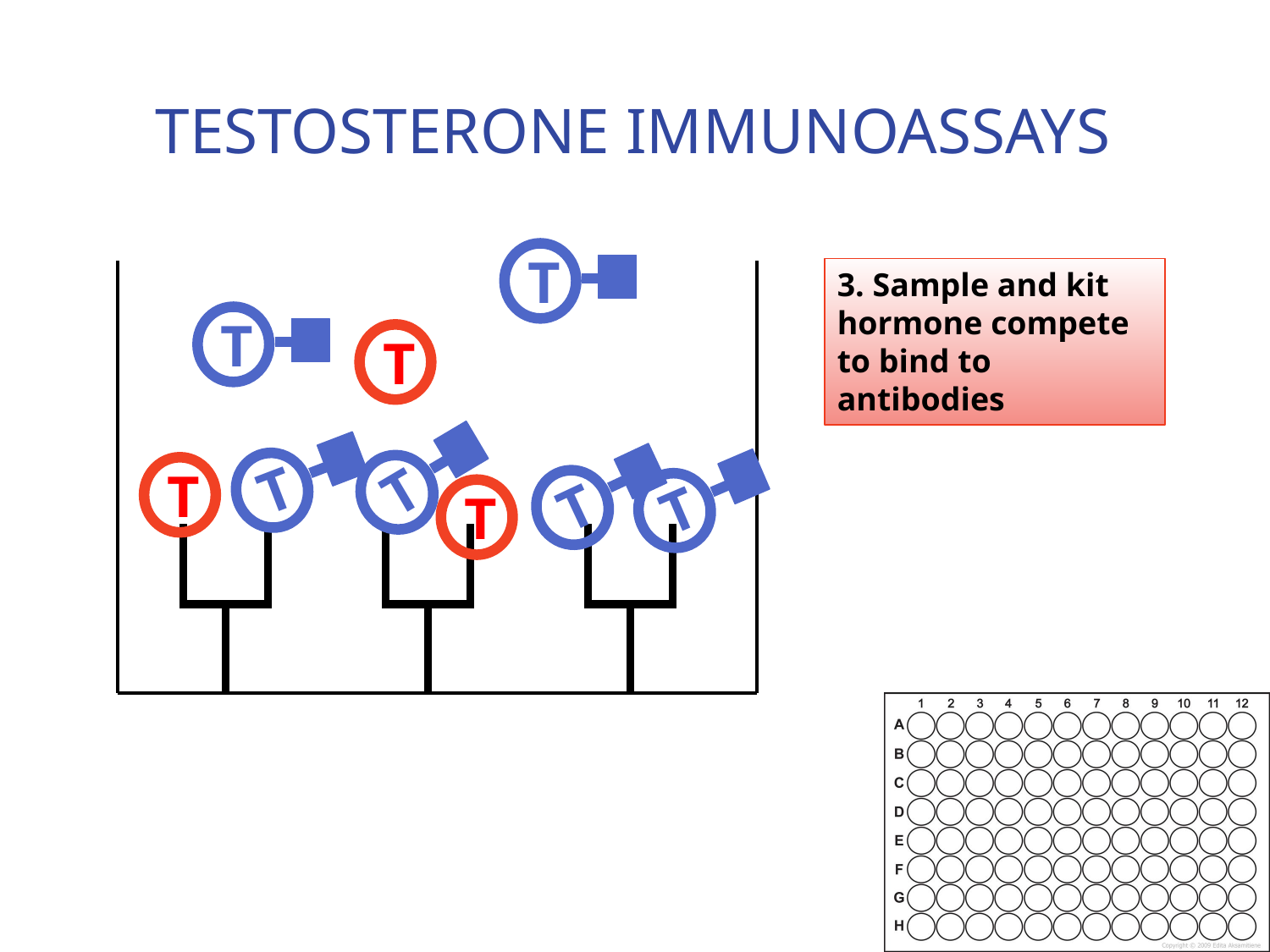

# TESTOSTERONE IMMUNOASSAYS
T
3. Sample and kit hormone compete to bind to antibodies
T
T
T
T
T
T
T
T

## Slide 6
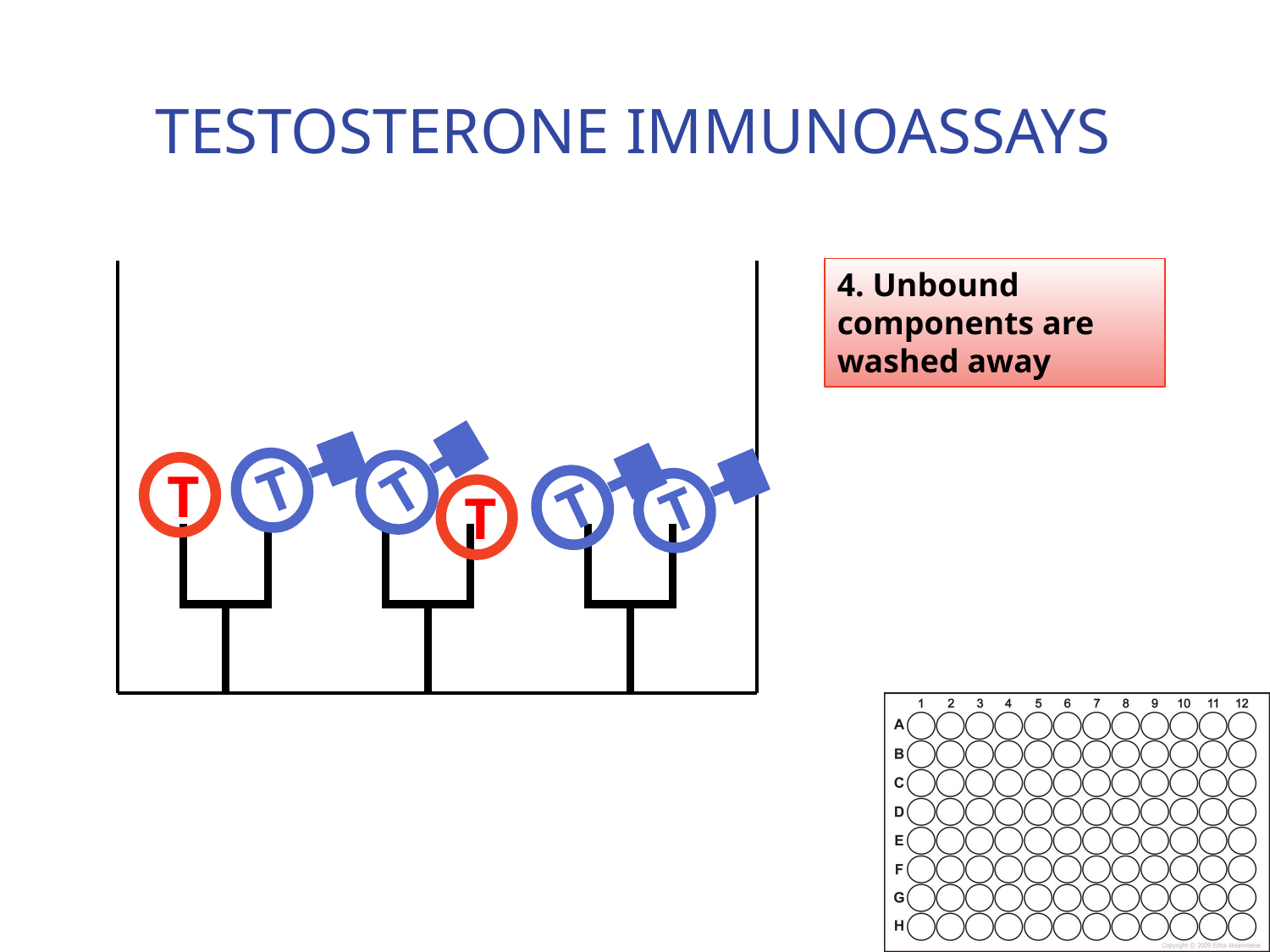

# TESTOSTERONE IMMUNOASSAYS
4. Unbound components are washed away
T
T
T
T
T
T

## Slide 7
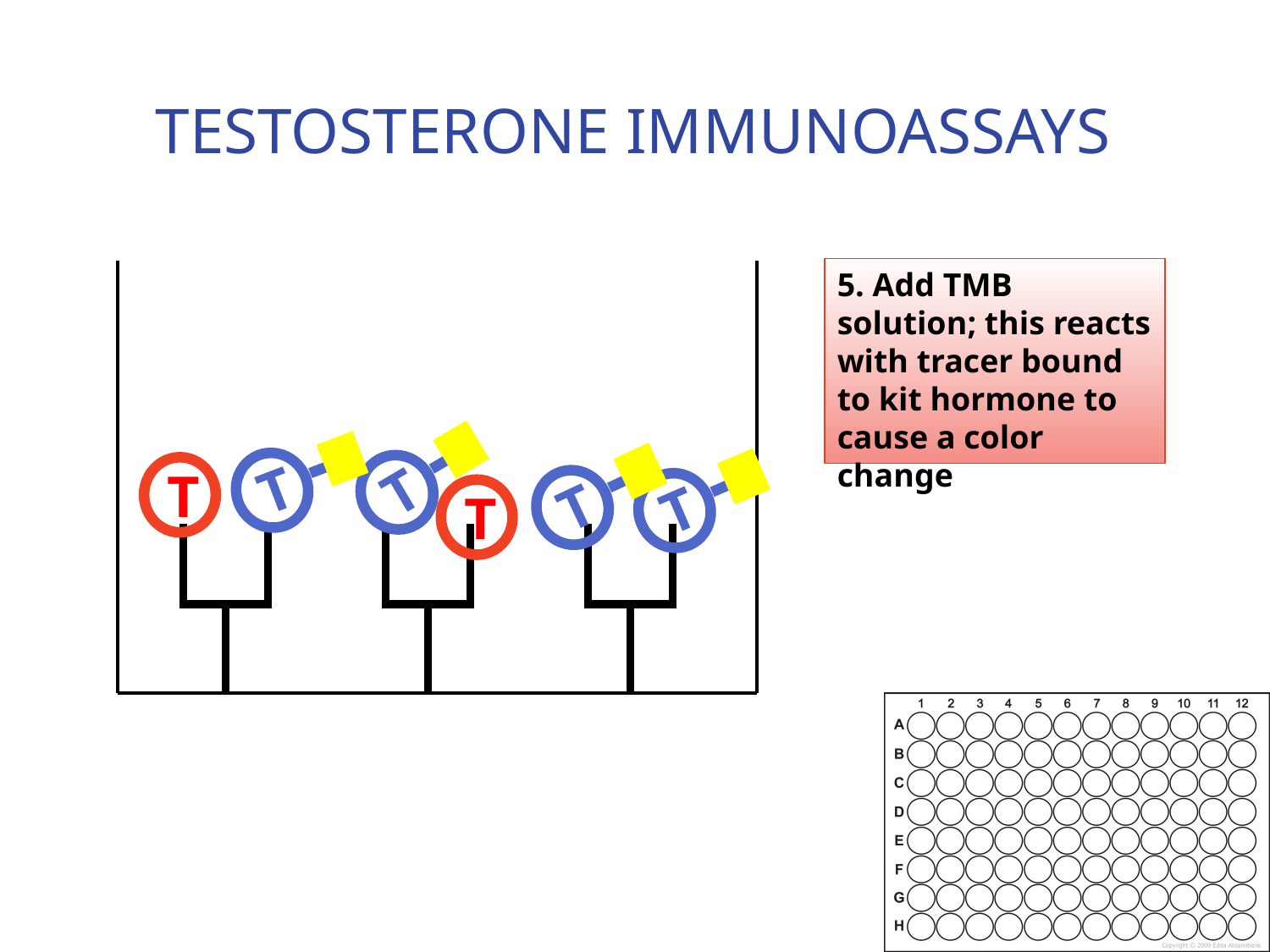

# TESTOSTERONE IMMUNOASSAYS
5. Add TMB solution; this reacts with tracer bound to kit hormone to cause a color change
T
T
T
T
T
T

## Slide 8
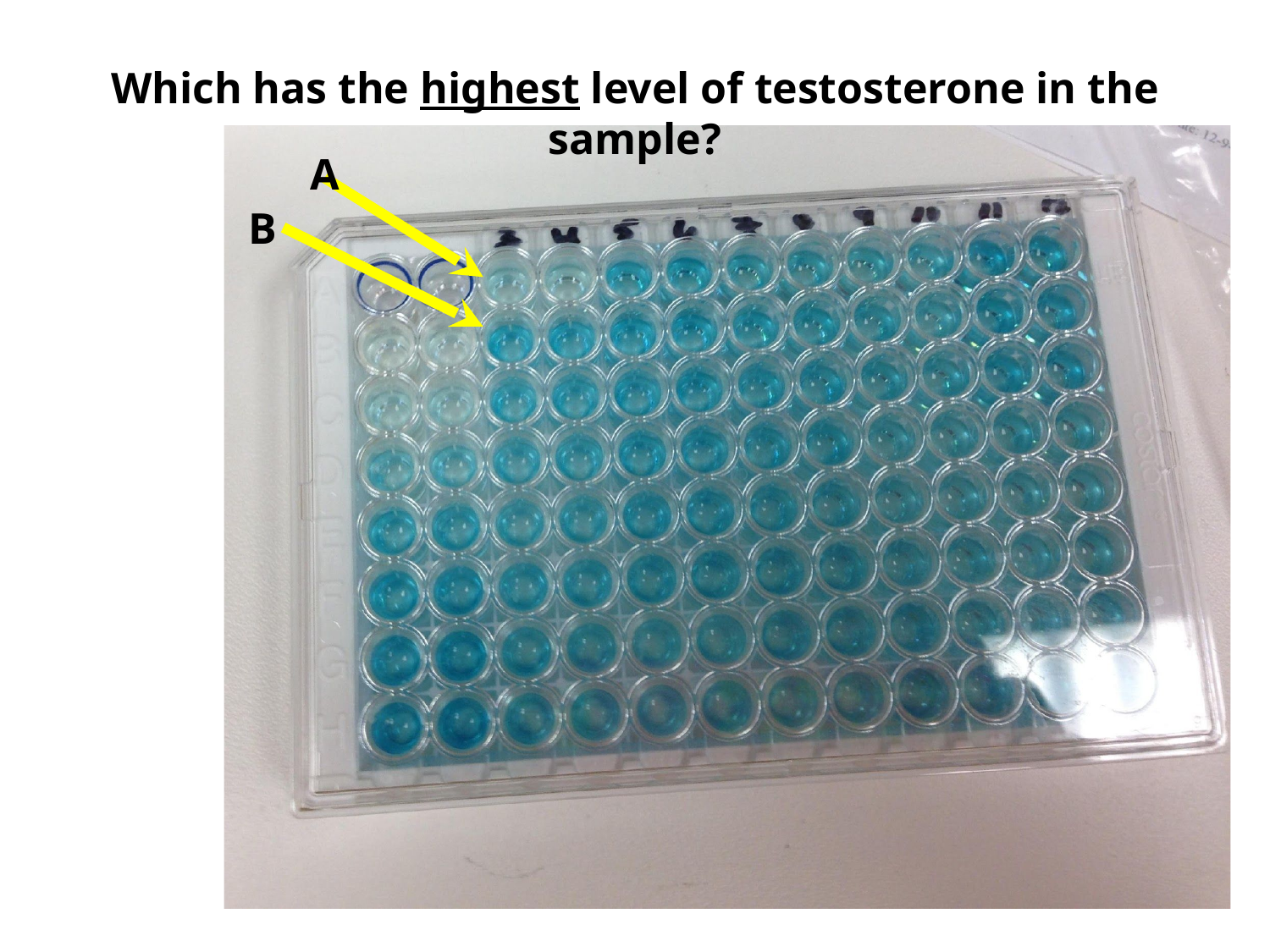

Which has the highest level of testosterone in the sample?
A
B

## Slide 9
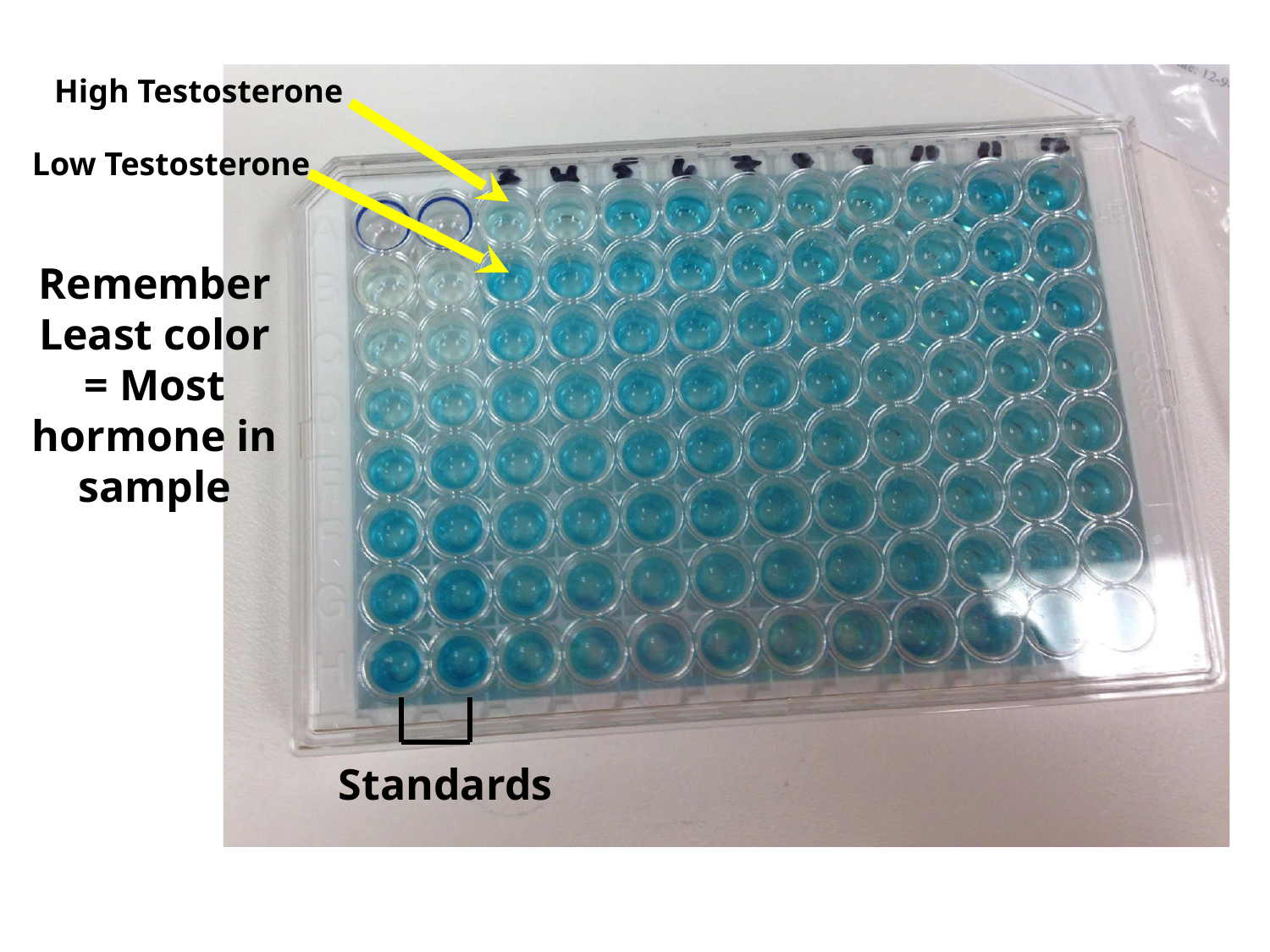

High Testosterone
Low Testosterone
Remember Least color = Most hormone in sample
Standards
