# Supplementary material for: Spit-Tacular Science: Collaborating With Undergraduates on Publishable Research With Salivary Biomarkers
Source: Front Psychol. 2019 Mar 21;10:562. doi: 10.3389/fpsyg.2019.00562 (PMC6437038; doi:10.3389/fpsyg.2019.00562)
Supplement: Supplementary file 2 [file Presentation_2.pptx]

## Slide 1
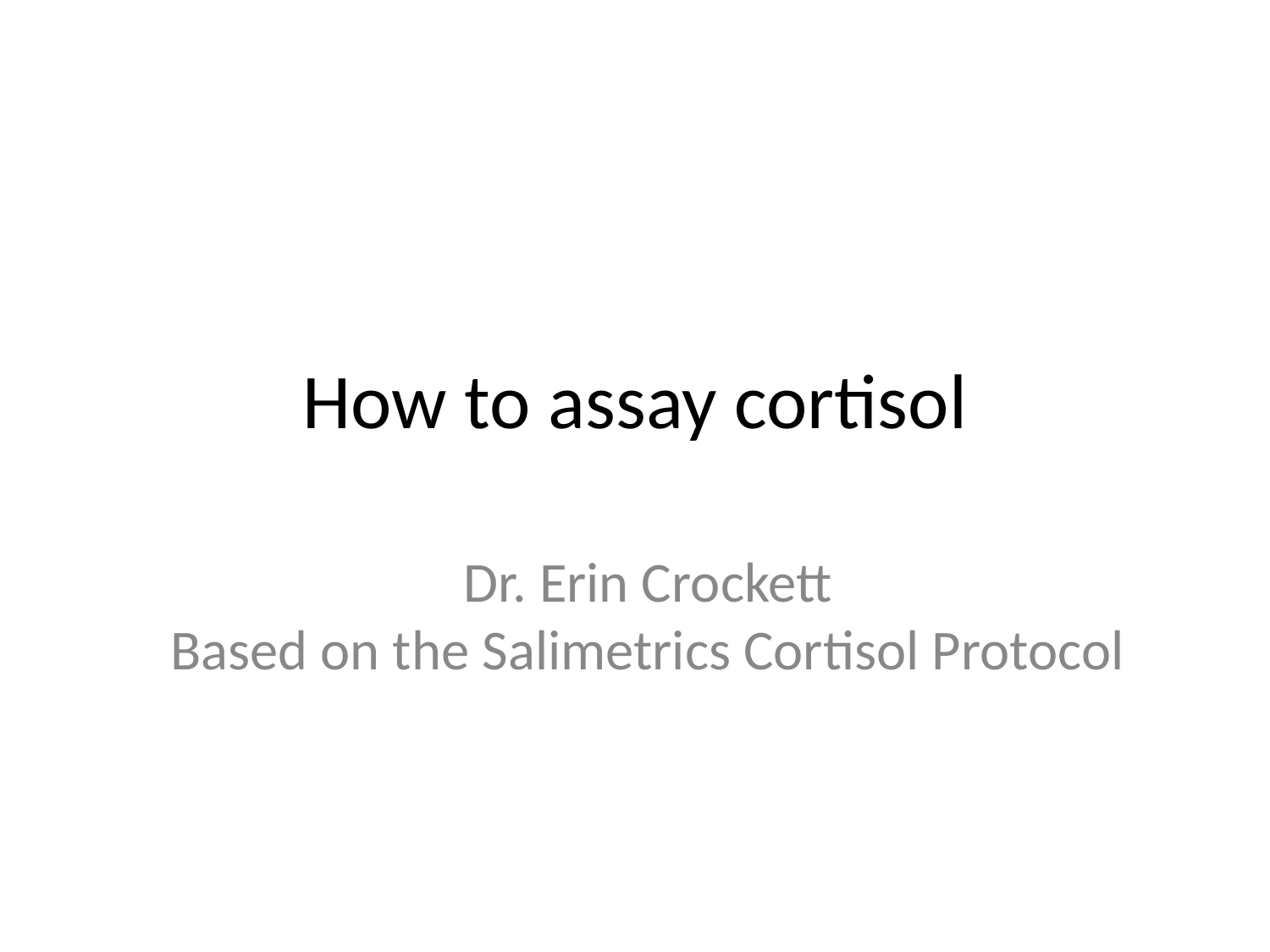

# How to assay cortisol
Dr. Erin Crockett
Based on the Salimetrics Cortisol Protocol

## Slide 2
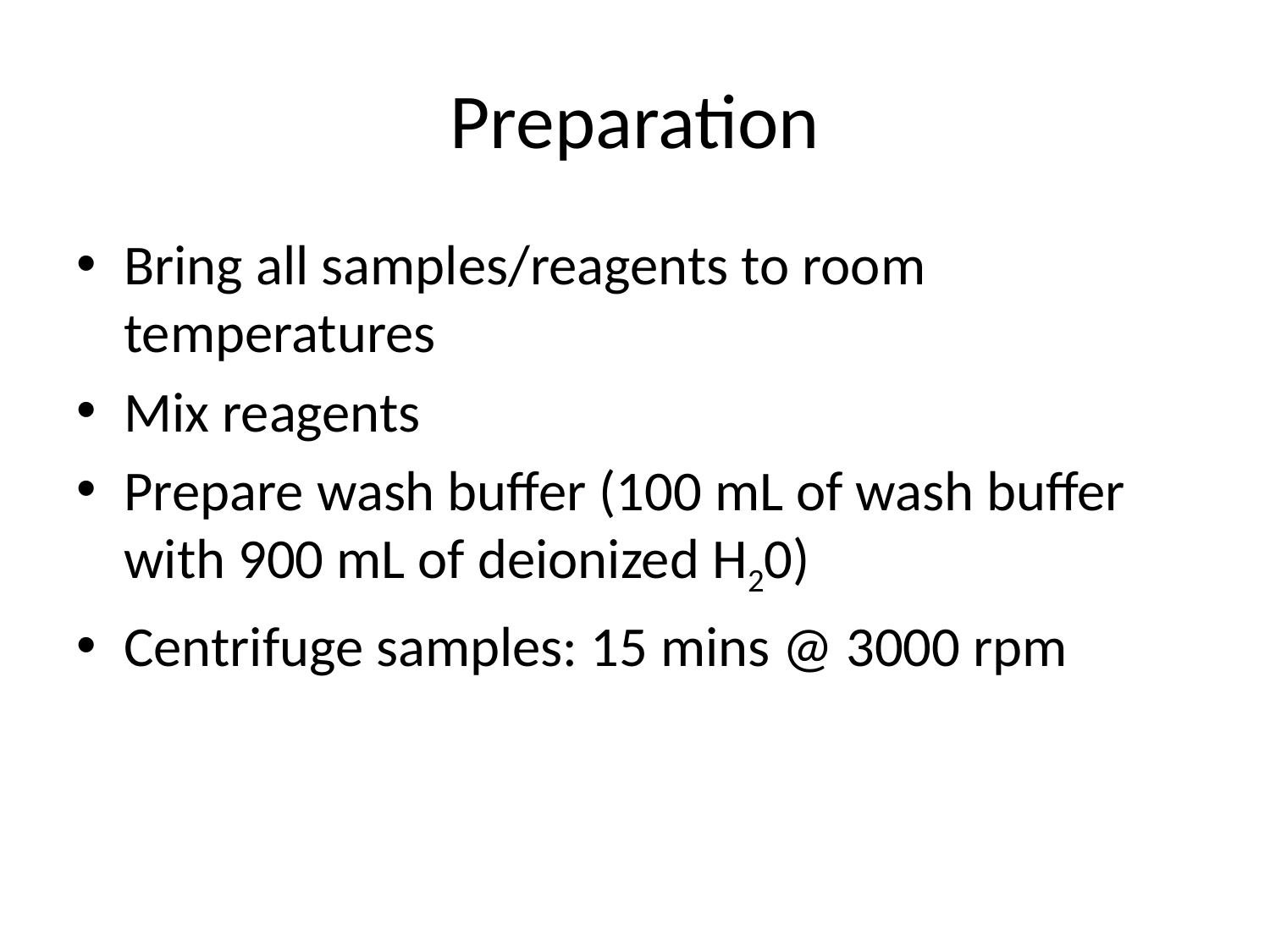

# Preparation
Bring all samples/reagents to room temperatures
Mix reagents
Prepare wash buffer (100 mL of wash buffer with 900 mL of deionized H20)
Centrifuge samples: 15 mins @ 3000 rpm

## Slide 3
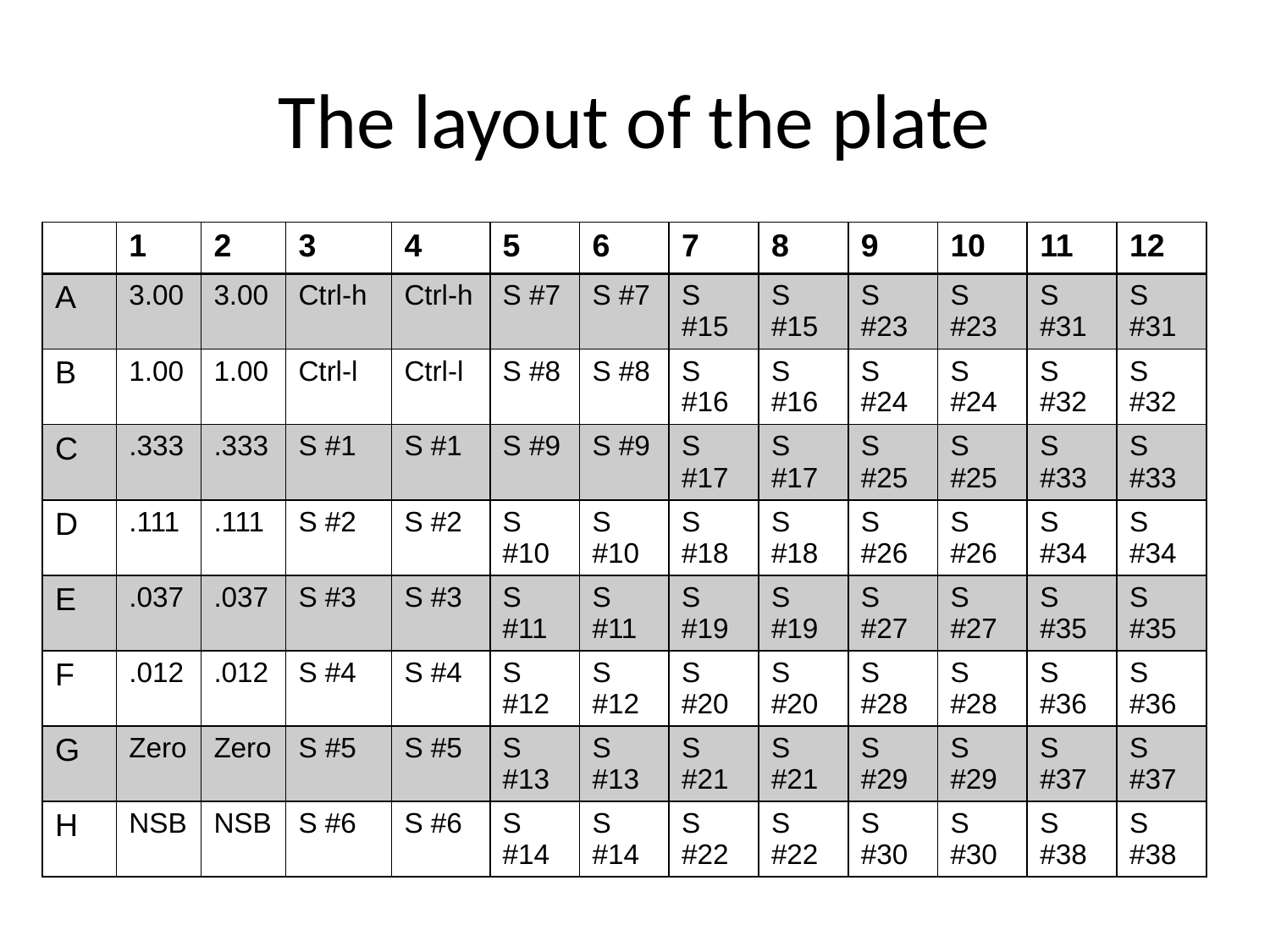

# The layout of the plate
| | 1 | 2 | 3 | 4 | 5 | 6 | 7 | 8 | 9 | 10 | 11 | 12 |
| --- | --- | --- | --- | --- | --- | --- | --- | --- | --- | --- | --- | --- |
| A | 3.00 | 3.00 | Ctrl-h | Ctrl-h | S #7 | S #7 | S #15 | S #15 | S #23 | S #23 | S #31 | S #31 |
| B | 1.00 | 1.00 | Ctrl-l | Ctrl-l | S #8 | S #8 | S #16 | S #16 | S #24 | S #24 | S #32 | S #32 |
| C | .333 | .333 | S #1 | S #1 | S #9 | S #9 | S #17 | S #17 | S #25 | S #25 | S #33 | S #33 |
| D | .111 | .111 | S #2 | S #2 | S #10 | S #10 | S #18 | S #18 | S #26 | S #26 | S #34 | S #34 |
| E | .037 | .037 | S #3 | S #3 | S #11 | S #11 | S #19 | S #19 | S #27 | S #27 | S #35 | S #35 |
| F | .012 | .012 | S #4 | S #4 | S #12 | S #12 | S #20 | S #20 | S #28 | S #28 | S #36 | S #36 |
| G | Zero | Zero | S #5 | S #5 | S #13 | S #13 | S #21 | S #21 | S #29 | S #29 | S #37 | S #37 |
| H | NSB | NSB | S #6 | S #6 | S #14 | S #14 | S #22 | S #22 | S #30 | S #30 | S #38 | S #38 |

## Slide 4
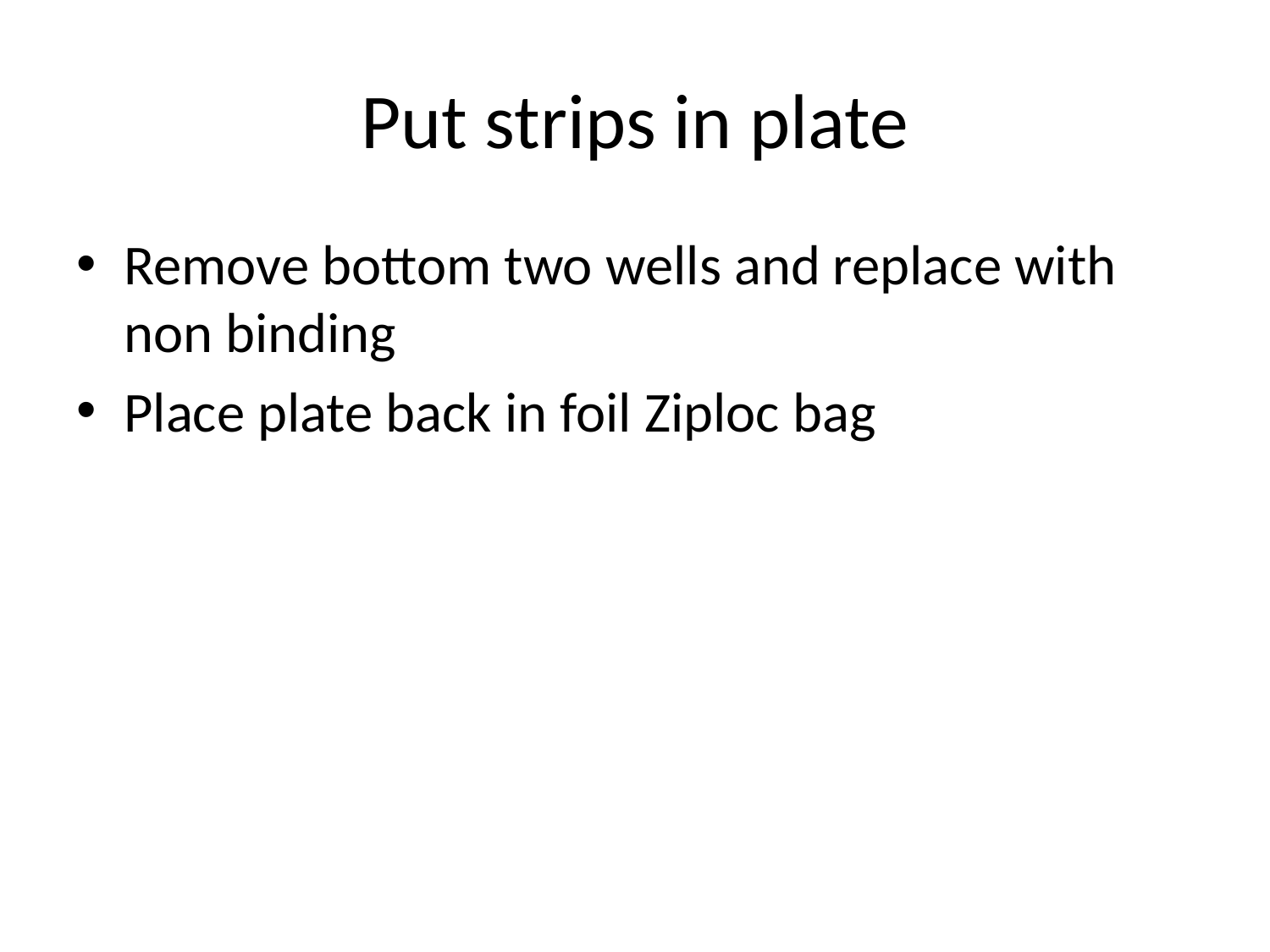

# Put strips in plate
Remove bottom two wells and replace with non binding
Place plate back in foil Ziploc bag

## Slide 5
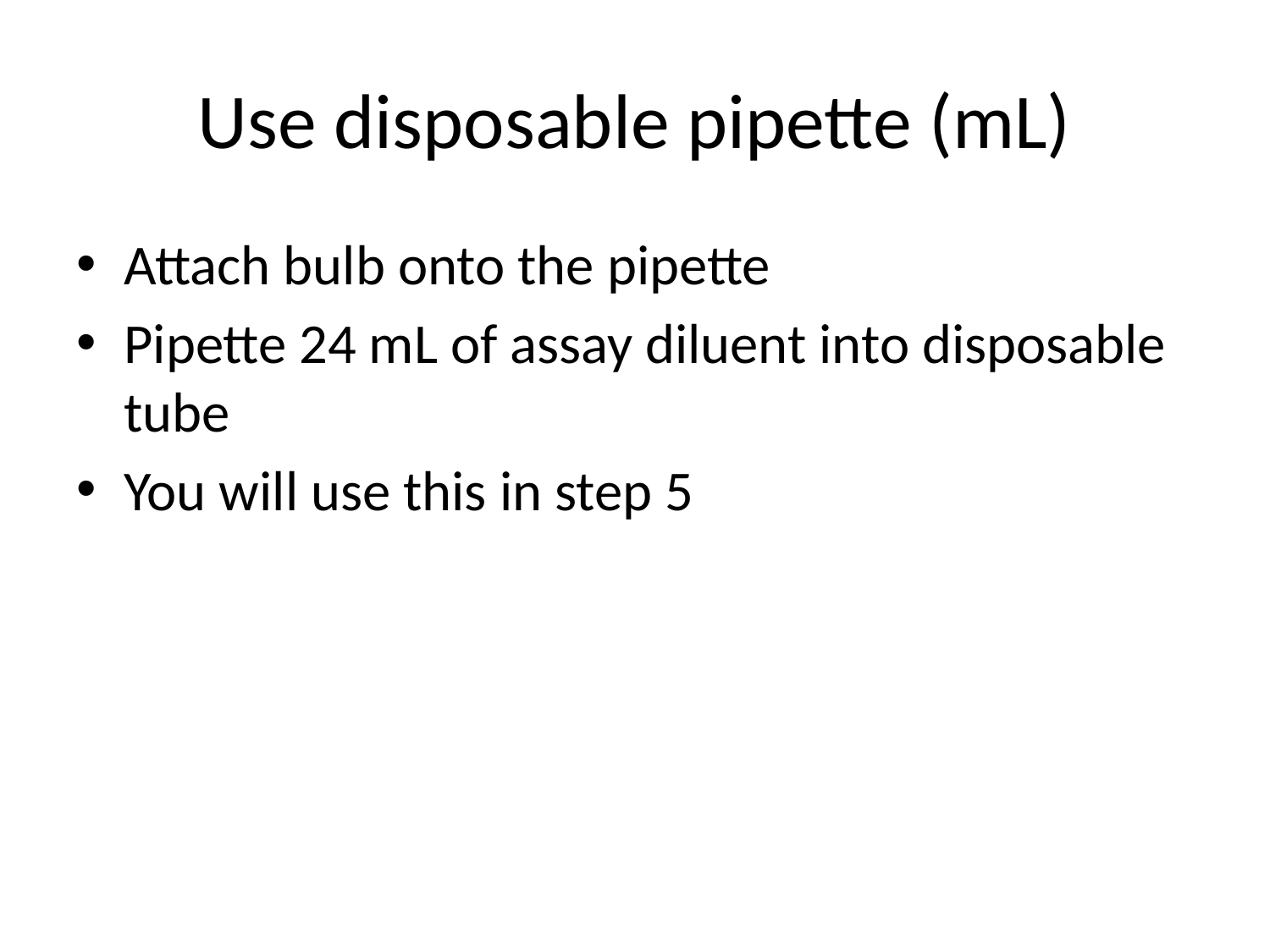

# Use disposable pipette (mL)
Attach bulb onto the pipette
Pipette 24 mL of assay diluent into disposable tube
You will use this in step 5

## Slide 6
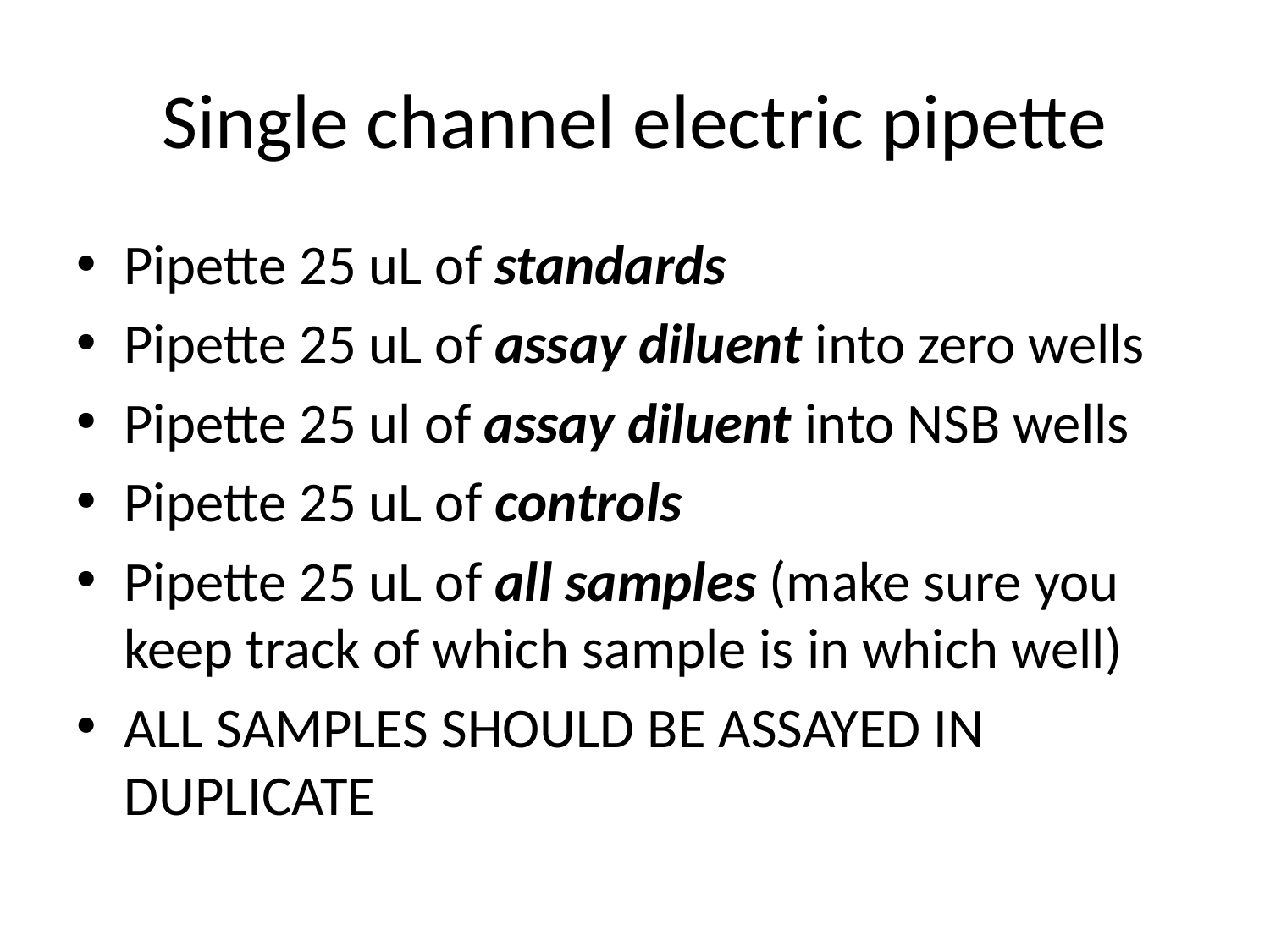

# Single channel electric pipette
Pipette 25 uL of standards
Pipette 25 uL of assay diluent into zero wells
Pipette 25 ul of assay diluent into NSB wells
Pipette 25 uL of controls
Pipette 25 uL of all samples (make sure you keep track of which sample is in which well)
ALL SAMPLES SHOULD BE ASSAYED IN DUPLICATE

## Slide 7
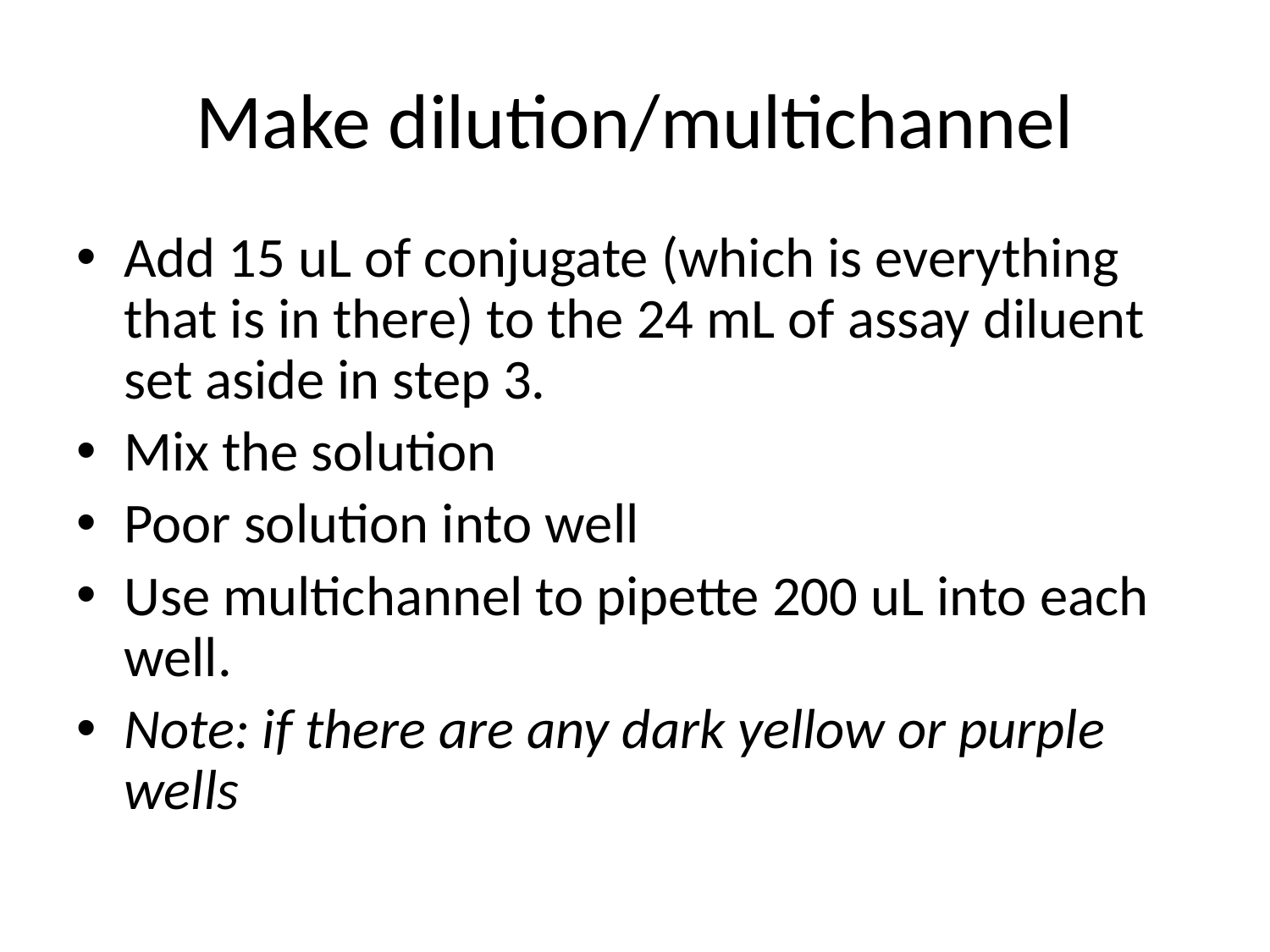

# Make dilution/multichannel
Add 15 uL of conjugate (which is everything that is in there) to the 24 mL of assay diluent set aside in step 3.
Mix the solution
Poor solution into well
Use multichannel to pipette 200 uL into each well.
Note: if there are any dark yellow or purple wells

## Slide 8
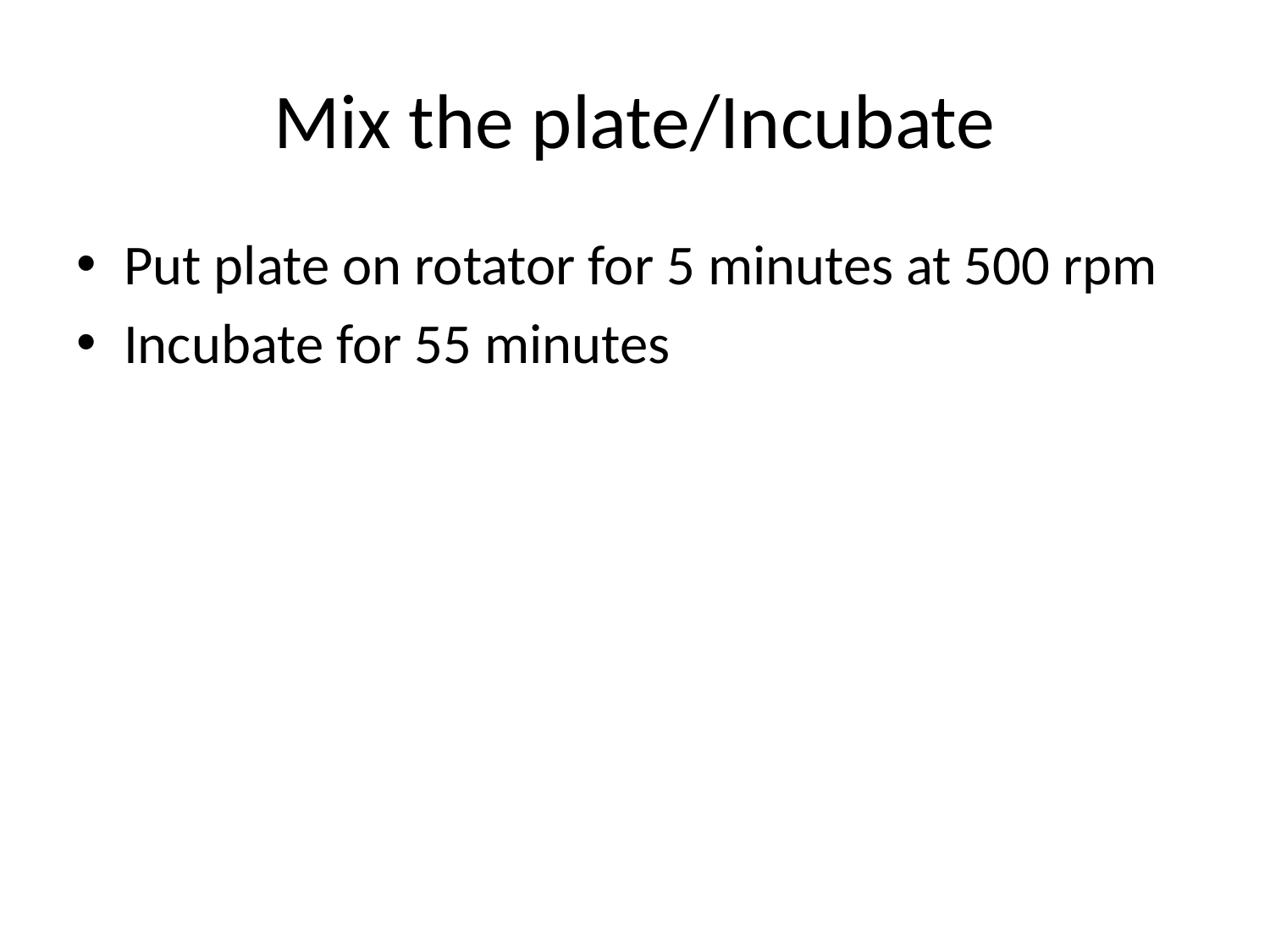

# Mix the plate/Incubate
Put plate on rotator for 5 minutes at 500 rpm
Incubate for 55 minutes

## Slide 9
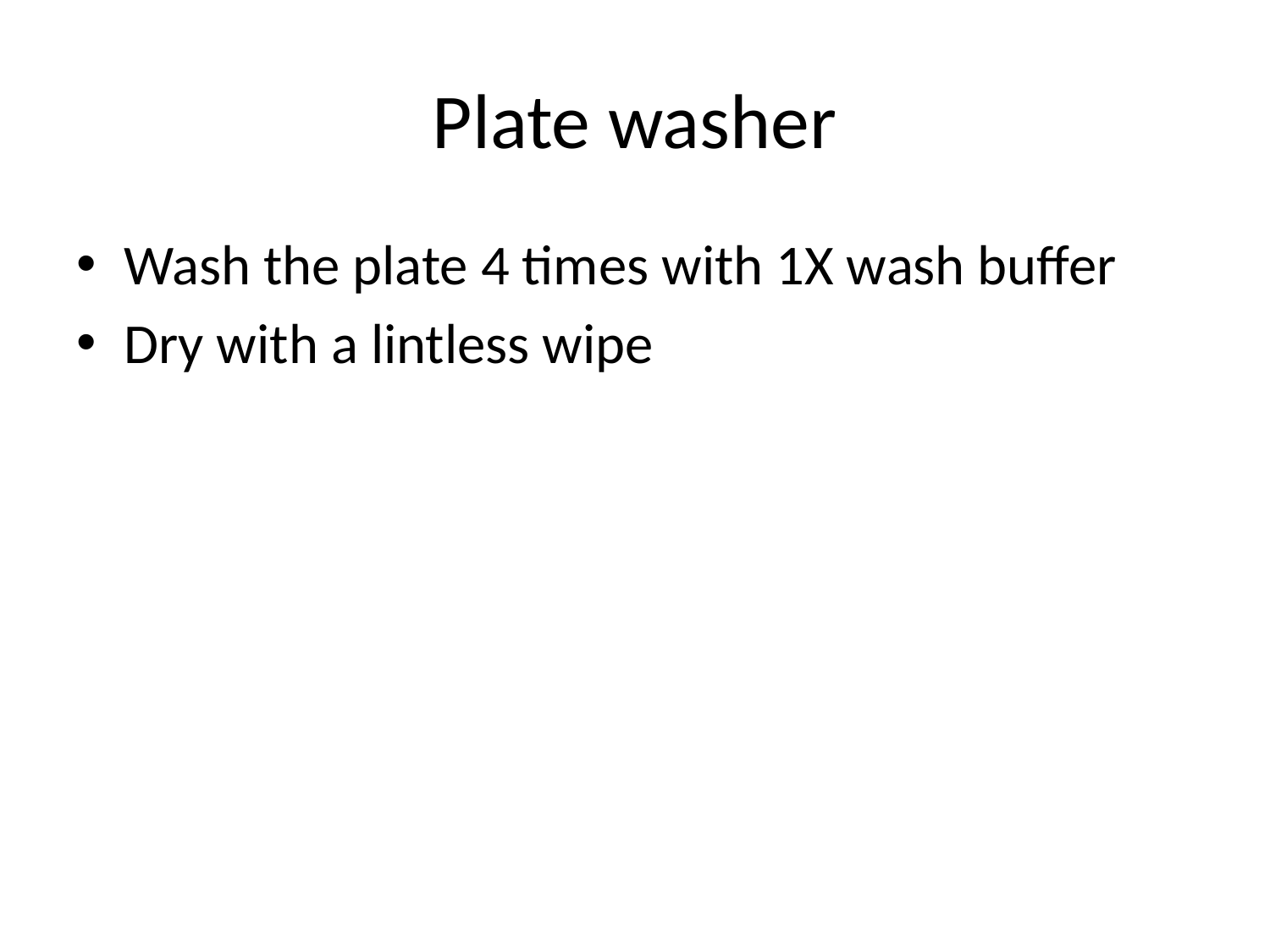

# Plate washer
Wash the plate 4 times with 1X wash buffer
Dry with a lintless wipe

## Slide 10
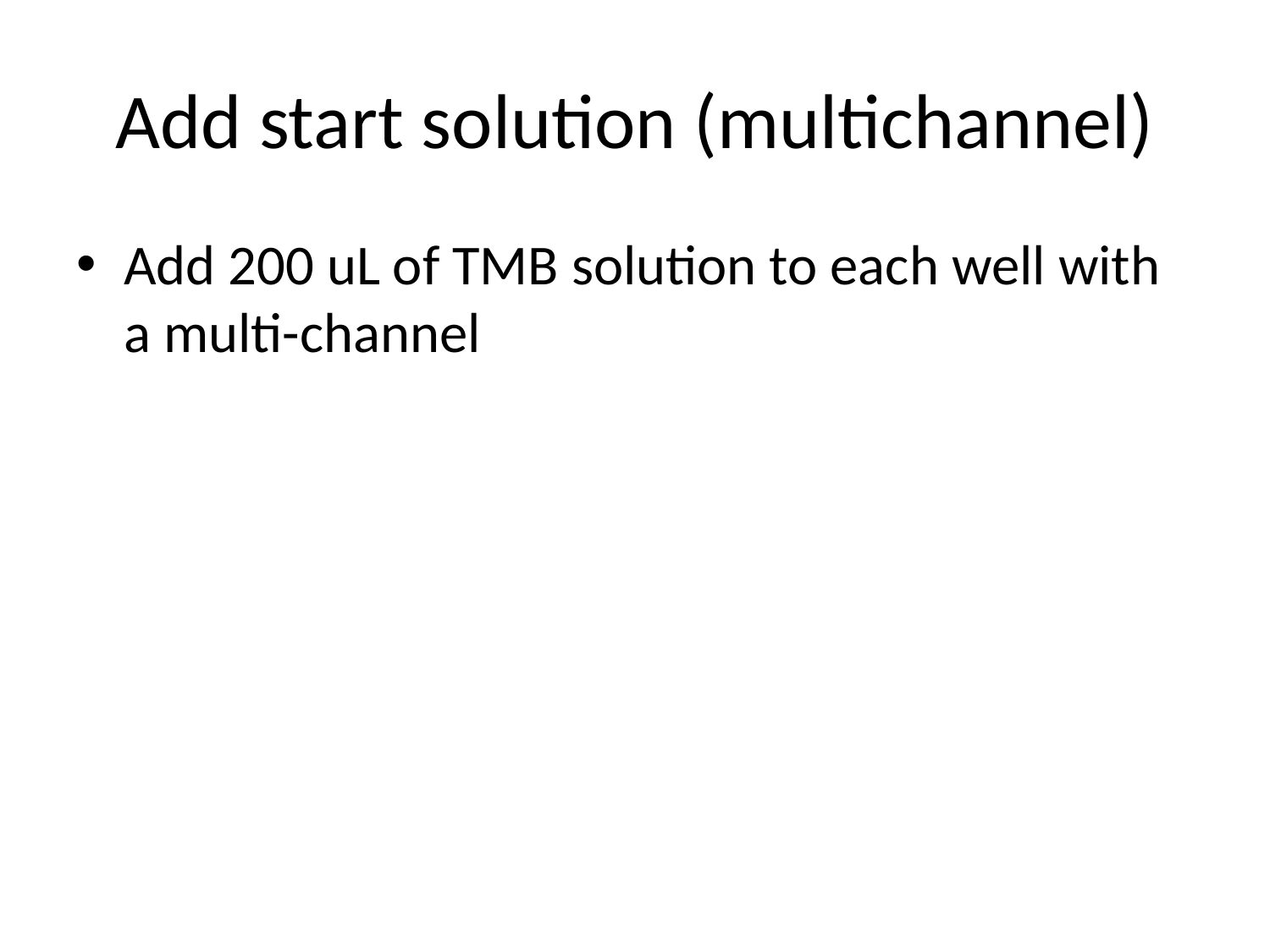

# Add start solution (multichannel)
Add 200 uL of TMB solution to each well with a multi-channel

## Slide 11
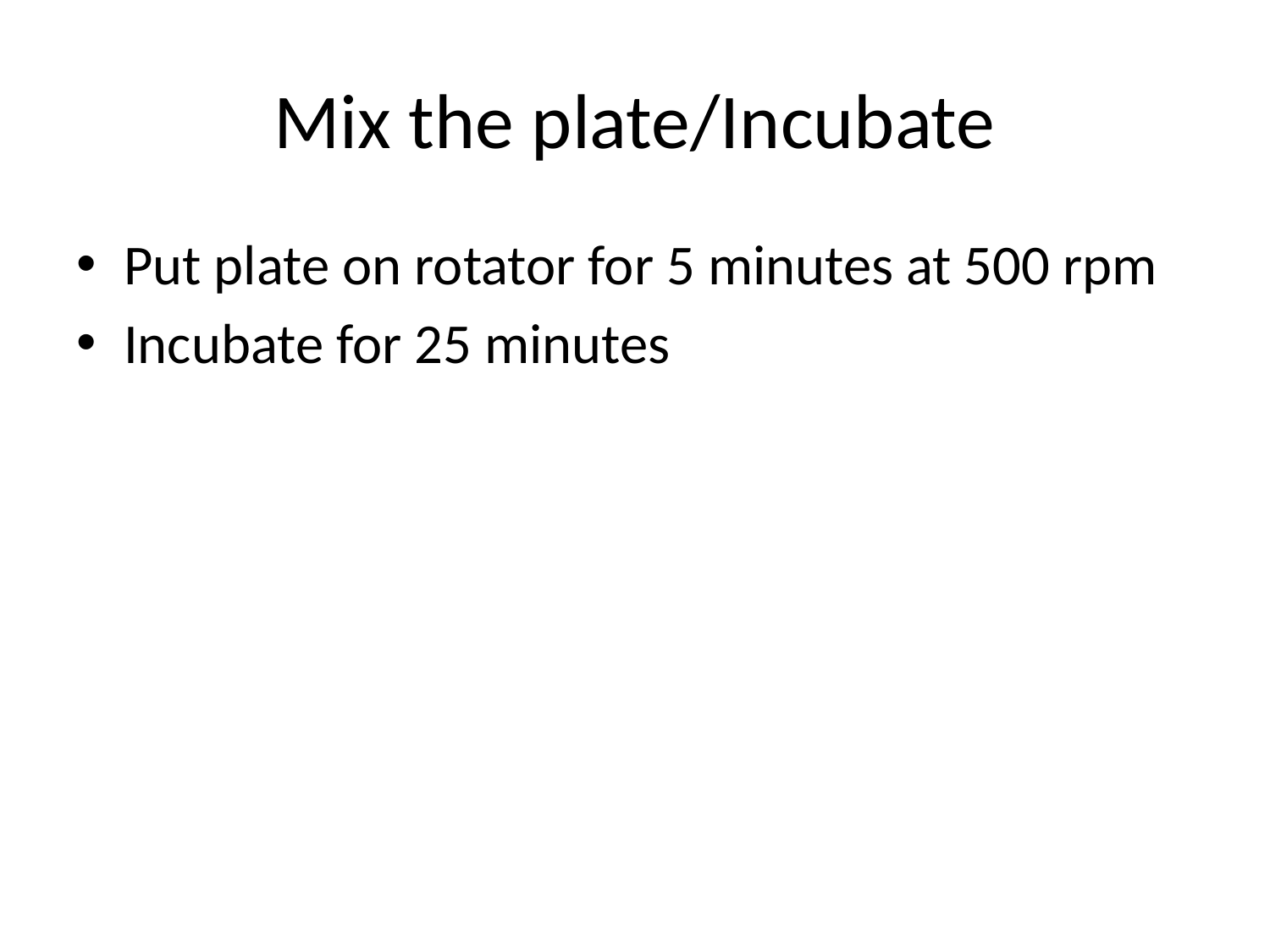

# Mix the plate/Incubate
Put plate on rotator for 5 minutes at 500 rpm
Incubate for 25 minutes

## Slide 12
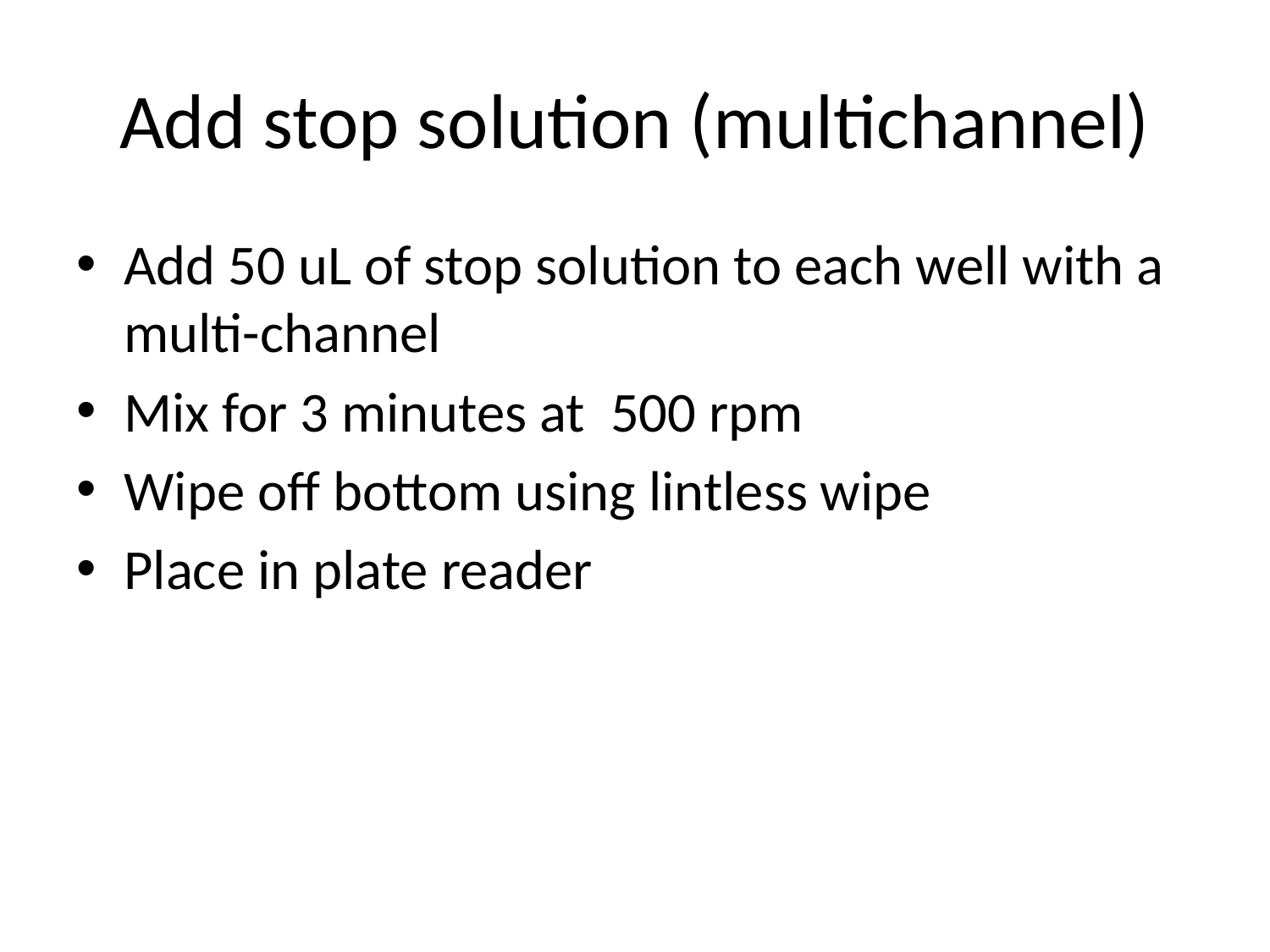

# Add stop solution (multichannel)
Add 50 uL of stop solution to each well with a multi-channel
Mix for 3 minutes at 500 rpm
Wipe off bottom using lintless wipe
Place in plate reader
