# Supplementary material for: Spit-Tacular Science: Collaborating With Undergraduates on Publishable Research With Salivary Biomarkers
Source: Front Psychol. 2019 Mar 21;10:562. doi: 10.3389/fpsyg.2019.00562 (PMC6437038; doi:10.3389/fpsyg.2019.00562)
Supplement: Supplementary file 3 [file Data_Sheet_1.docx]

Saliva Collection Instructions for Research Participants: Passive Drool

1. Please use a tissue to wipe the outside of your mouth (to remove any chapstick, etc.).
2. Take a sip of water, swish, and swallow the water to rinse your mouth.
3. Put your bottom lip against the tube, and as saliva gathers in your mouth spit it into the tube.
4. Aim to reach the 4mL line marked on the tube.
   1. If you are struggling to produce enough saliva or it is taking you a long time (more than 10 minutes), you can aim for 2-3mL.
   2. Sometimes saliva is frothy and bubbly. If possible the clear fluid should be up to the marked line.
5. Reminders:
   1. Do not touch the inside or top of the tube.
   2. Do not drink anything back from the tube.
   3. Do not pour anything out of the tube. When you are finished with your sample just set it upright in the cup.
   4. You are welcome to use a tissue if you would like to wipe your mouth or the outside of the tube at any time.

*Note. Standard ELISA techniques, per the company’s protocols, should be followed for analyzing the biomarker in question.*
