# Supplementary material for: Spit-Tacular Science: Collaborating With Undergraduates on Publishable Research With Salivary Biomarkers
Source: Front Psychol. 2019 Mar 21;10:562. doi: 10.3389/fpsyg.2019.00562 (PMC6437038; doi:10.3389/fpsyg.2019.00562)
Supplement: Supplementary file 4 [file Data_Sheet_2.docx]

Cortisol Procedures

Dr. Erin Crockett

**How do we collect samples?**

Saliva samples are a non-invasive way to assess cortisol that involves participants putting a 2-inch piece of dental cotton in their mouths for 2 minutes. This procedure is very safe for participants and is now commonly used in psychological research. Saliva samples will be collected via use of the Salivette^®^ – a small, rolled piece of cotton on which participants are asked to lightly chew for two minutes (see figure below). The Salivette^®^ is then placed in a plastic tube and later centrifuged to separate the saliva from the cotton. This method of saliva collection has been used in hundreds of studies, requires minimal disruption, and is non-invasive (e.g., relative to collecting plasma samples). A dry mouth is the most common complaint of participants undergoing this procedure.


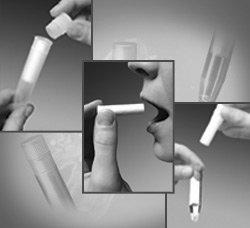


Script:

*We will be taking saliva samples regularly with a device called a salivette, about every 15 minutes throughout the study. To do this, I will give you a piece of sterile dental cotton and ask you to place it in your mouth for about two minutes.*

Pop open top of salivette and let participant see it. Don’t touch the cotton.

*Let’s go ahead and take your first sample now so you can become familiar with the process. Just place this piece of cotton in your mouth and I’ll let you know when you can take it out. To get the cotton out, you can either tip the tube upside down directly in your mouth, or tip it into your hand and then place it in your mouth; whatever is more comfortable for you. You don’t want to chew on the cotton but you can make a chewing motion to help generate more saliva. The key is to get the sample very wet.*

Collect sample after 2 minutes.

**How do we store samples?**

All samples are stored in a -20 degree Celsius freezer. Samples are labeled by participant ID and sample number (e.g., 1-4). They are not identified by any personal information about the participant (e.g., name).

**How do we handle samples?**

All samples are assayed by the principal investigator, often with students assisting in the assays. We typically use Salimetrics cortisol assay kits. More information about the procedures required for these kits can be found here: <https://salimetrics.com/wp-content/uploads/2018/03/salivary-cortisol-elisa-kit.pdf>. Standard lab safety procedures are followed, including the wearing of lab coats, closed toed shoes, gloves and eye protection.

**How do we dispose of samples?**

Once samples have been assayed, they are refrozen until appropriate analysis confirming good reads can be confirmed. After this, all samples are thrown away in a laboratory trashcan.
